# Reshaping the role of m6A modification in cancer transcriptome: a review

**DOI:** 10.1186/s12935-020-01445-y

**Published:** 2020-07-29

**Authors:** Guanqun Yang, Zhigang Sun, Nan Zhang

**Affiliations:** 1 Department of Oncology, Jinan Central Hospital Affiliated to Shandong University, Cheeloo College of Medicine, Shandong University, Jinan, 250013 Shandong China; 2 Department of Thoracic Surgery, Jinan Central Hospital Affiliated to Shandong University, Cheeloo College of Medicine, Shandong University, Jinan, 250013 Shandong China

**Keywords:** m6A, RNA modification, Transcriptome, Posttranscriptional regulation, Cancer

## Abstract

N6-methyl-adenosine(m6A) modification emerges as an abundant and dynamic regulation throughout the Eukaryotic transcriptome. Dysregulation of the m6A regulators has increasingly been found in many neoplasms. It is reasonable to believe that m6A changes the fate of cancer cells and subsequently affected all aspects of cancer progression. In view of the context-dependent role of m6A modification, we emphasize a dual effect of m6A in a particular tumor model, that is, m6A plays a promoting role or a suppressing role in different stages of cancer. This novel sight is compared to the older view that a particular m6A regulator acts as a consistent role in cancer progression.

## Background

m6A modification is the most abundant eukaryotic RNA modification and can regulate many essential biological processes of the transcriptome, such as RNA splicing, nuclear export, stability, translation, and decay. m6A modification in human tumor cells has a significant influence on tumorigenesis, stemness, proliferation, invasion, metastasis, and response to immune via regulation of many oncogenes and tumor suppressor genes. Therefore, control of m6A modification in tumor cells is a potential anticancer therapeutic target.

**Fig. 1:**
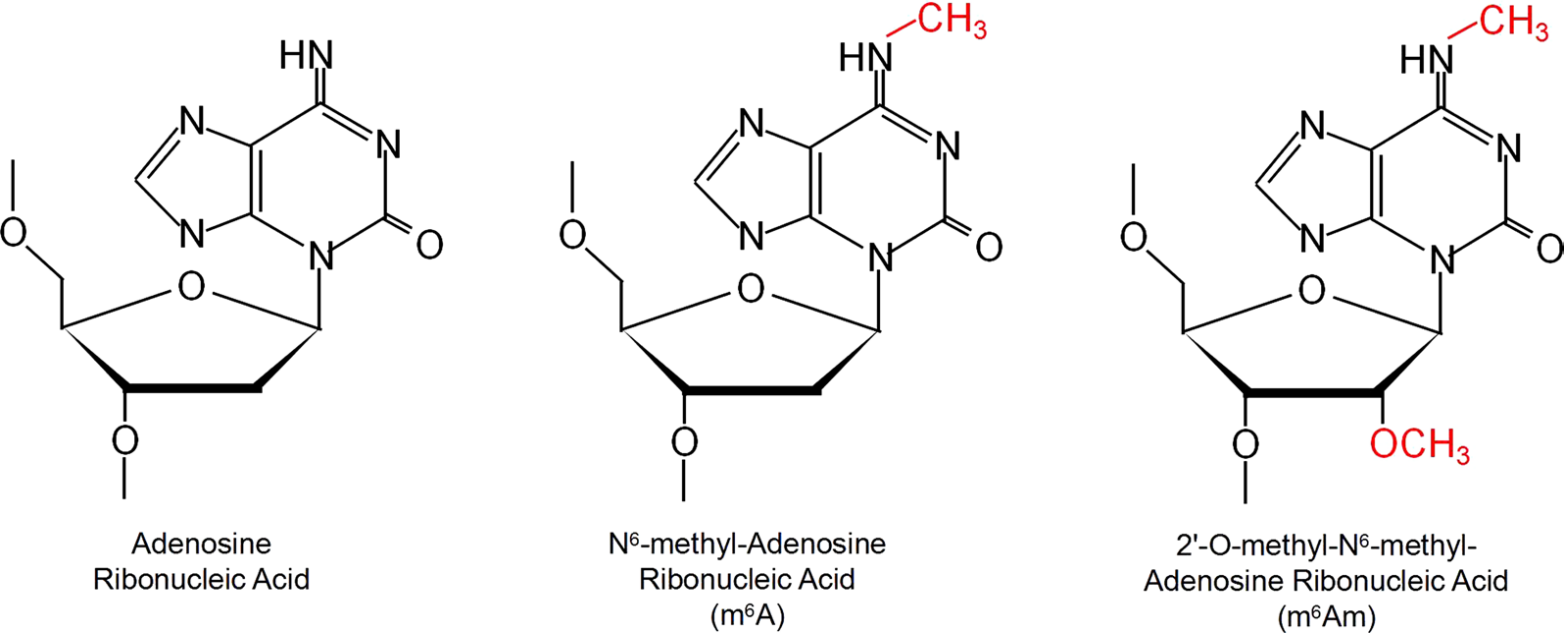
Chemical structure of adenosine modification

m6A was first detected in a poly(A)-mRNA fraction as a mode of mRNA chemical modification regulation in 1974 [1]. However, there has been no gratifying progress on the study of m6A due to lack of methods for detecting m6A sites in transcriptome. Researchers were not able to identify the individual m6A-RNA site and it was tough to distinguish m6A from adenosine (A) or 2′-O-methyl-N6-methyl-Adenosine Ribonucleic Acid (m6Am) (Fig. 1). m6Am is able to react with m6A-specific antibody m6Am contains the same N6-methyl as m6A on the basis of 2′-O-methyl-adonesine.

The innovation and development of high-throughput sequencing have significantly improved the above situation. By combining highly specific m6A antibodies and high-throughput sequencing, two scientific groups independently developed the first transcriptome-wide m6A sites mapping method termed Methylated RNA Immunoprecipitation Sequencing (MeRIP-Seq) in individual RNAs [2, 3]. This method could identify tens of thousands of candidate m6A modification sites at an average resolution of 100 nucleotides. Liu et al. developed a low-throughput method termed Site-specific Cleavage And Radioactive-labeling followed by Ligation-assisted Extraction and Thin-layer chromatography (SCARLET) that could detect the presence of the particular modification fractions [4]. In addition, m6A individual-nucleotide-resolution Crosslinking and Immunoprecipitation (miCLIP) makes single-nucleotide-resolution mapping possible [5].

These technological advances have revealed many novel characteristics and mechanisms of this pervasive transcriptome modification. Researchers surprisingly found that m6A is highly selective: [1] Only some of RNAs contain m6A [2]; 2 m6A usually appears near stop codon and in 3′UTR [3]; 3 The DRACH (D = G, A, or U; R = G or A; and H = C, A, or U) motif is a consensus sequence of the m6A modification [6, 7]. The level of m6A in transcriptome was determined to be dynamic, varying in cell development and response to stresses [2, 3].

**Fig. 2:**
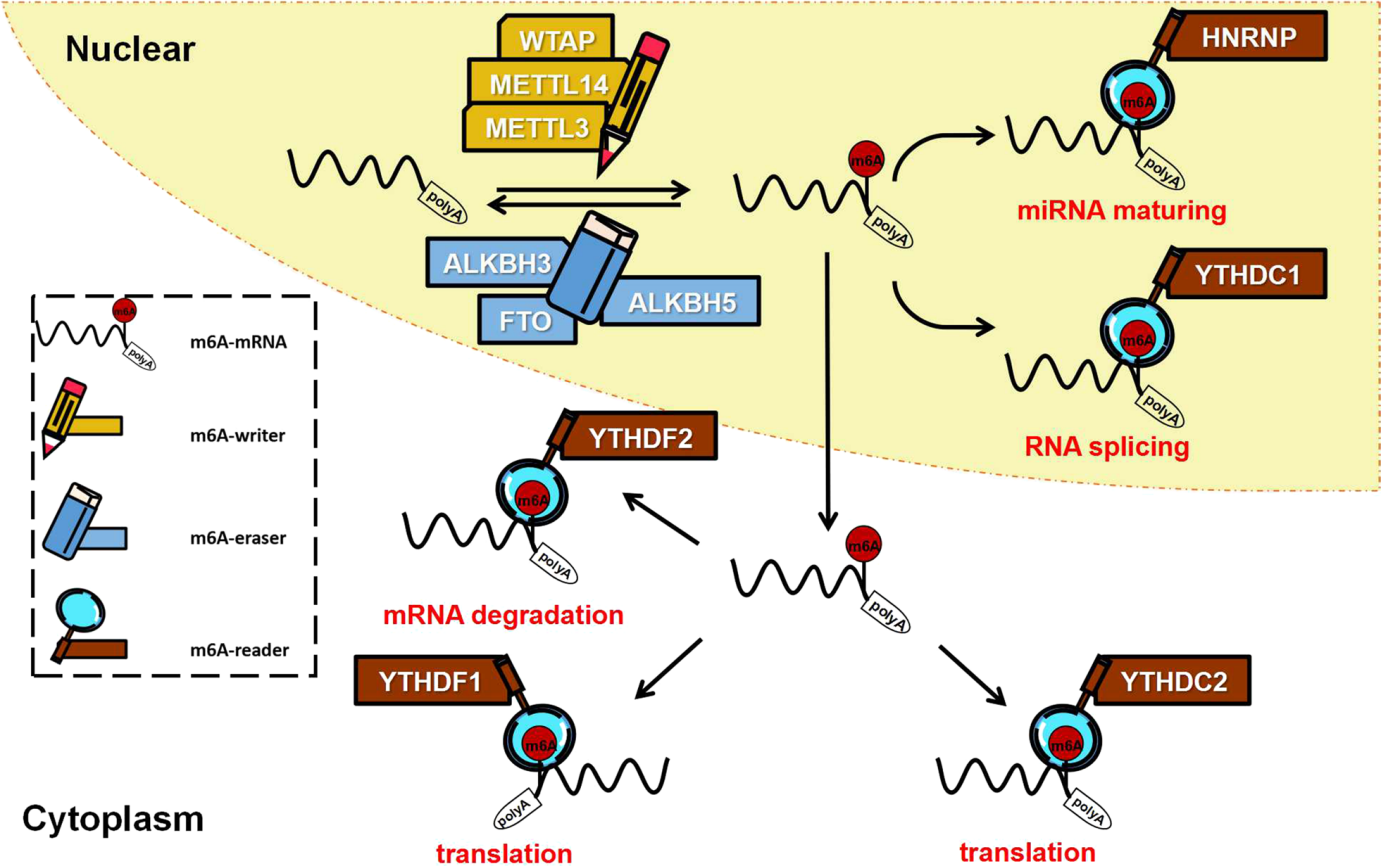
Simplified model of m6A Dynamic regulation

The idea that adenosine N6-methylation is reversible provides the foundation of the m6A dynamic regulation hypothesis. N6 sites of RNA adenosine can be methylated by special methyltransferase, such as Methyltransferase-like 3 (METTL3) [8, 9]. Various kinds of RNA binding proteins exist in Eukaryotic cells. Some of them can recognize and decode m6A, then change the structures of modified RNAs, and finally determine the fates of m6A-RNAs. Among them, what we know most about is the YTH domain protein family [10]. In the past few years, other m6A binding proteins have been discovered. Some results of studies on how these m6A binding proteins work and what they do are controversial or even opposite. m6A can be removed by demethylase, like fat mass and obesity-associated protein (FTO) and Alpha-ketoglutarate-dependent dioxygenase (ALKB) homolog 5 (ALKBH5) [11, 12]. Controversies also exist regarding the effects of RNA demethylases, which we describe in detail below. Thus, ‘writer’ adding m6A modification, ‘reader’ decoding m6A modification and embodying its roles, and ‘eraser’ exerting demethylation activity form a network of m6A dynamic regulation (Fig. 2). Once the m6A “Reader-Writer-Eraser” model was proposed, it has become the core of the m6A study. However, we still know very little about the newly discovered m6A regulators, and many gaps lie in our understanding of the dynamic and selective regulation mechanism of m6A. Therefore, improved understanding of the m6A “Reader-Writer-Eraser” model and its working mechanism in human cancer should lead to novel anticancer strategy.

With the deepening understanding of m6A, it is hard to simply categorize an m6A regulator into an oncogene or tumor suppressor in different cancer models. For instance, the eraser FTO may act as an oncogene in lung carcinoma [13] and as a tumor suppressor in renal clear cell carcinoma [14]. Also certain protein may have dual effects in different progression stages of an exclusive tumor model. One example is that YTHDF2, an m6A reader, may promote the proliferation of pancreatic cancer cells, whereas it suppresses metastasis in pancreatic cancer [15]. Arguments on the role of m6A modification in transcriptome hinder the application of this important posttranscriptional regulation to cancer diagnosis and treatment, hence reshaping the role of m6A modification in human cancer becomes crucial.

This review is to provide the current view of m6A regulating transcriptome dynamically and selectively through the novel regulatory model, and facilitate a better understanding of the complex effects of m6A modification on the transcriptome of human cancer cells.

## m6A writer

Rottman et al. first discovered and purified the first m6A methyltransferase, now referred to as METTL3 [8, 9]. Subsequently, other suspected methyltransferases were discovered, such as Methyltransferase-like 14 (METTL14) and Wilms’ tumor 1-associated protein (WTAP), although finally confirmed that they had no methyltransferase activity. The latest sight is that it is METTL3-METTL14-WTAP methyltransferase complex that is located to nuclear speckles and responsible for m6A formation.

S-adenosyl methionine (SAM) is an important auxiliary substrate involved in transmethylation. METTL3 is a member of SAM-dependent methyltransferase family, which widely exist in eukaryotic cells and is highly conserved in mammals [16]. Genetic deletion of METTL3 results in nearly complete loss of m6A in A-rich RNA [17–19]. Thus, METTL3 is the main m6A methyltransferase of eukaryotic poly-A mRNA. METTL3 does not contribute to m6A formation of rRNA and small nuclear RNA (snRNA) [20]. Therefore, novel m6A writers remain to be discovered to fill this gap.

Once it was discovered, METTL14 was regarded as the second methyltransferase enzyme [21]. However, three separate crystallization studies showed that METTL14 lacks a SAM-binding domain and that purified METTL14 does not have any methyltransferase activity [22–24]. Previous misunderstandings of data are considered to be due to METTL3 coprecipitation. Still, METTL14 does is instrumental in enhancing catalytic activity, as well as positioning the methyl group [22–24].

WTAP works as an adaptor protein without catalytic activity for m6A modification [25]. WTAP depletion causes loss of METTL3 and METTL14 in nuclear speckles and loss of m6A formation in mRNA. Thus, WTAP maintains METTL3 location. It is caused by the lack of the nuclear localization sequence (NLS) [26].

METTL3, METTL14, and WTAP form the m6A methyltransferase complex. METTL3, as the catalytic core, transfers the methyl group from the SAM to the RNA. METTL14 serves as the RNA-binding arm, promoting the affinity to the RNA substrate and enhancing the complex integrity [22, 27]. METTL3 and METTL14 form a stable asymmetric heterodimer, which then binds to WTAP [28]. WTAP assists m6A methyltransferase complex to localize in nuclear speckles [28].

## m6A eraser

Ever since discovering m6A methyltransferase, people have been working hard to realize the idea of m6A dynamic regulation through searching enzymes that remove m6A methyl groups. Situation had been very upset until the discovery of the first m6A associated demethylase FTO in 2011 [11]. However, increasing evidences are pointing towards the idea that FTO acts on m6Am rather than m6A. Nevertheless, we will introduce it below. Another important demethylase is ALKBH5 [12]. Both FTO and ALKBH5 belong to the Fe^2+^-α-ketoglutarate-dependent dioxygenase family. Recent research found a new demethylase, ALKBH3, which prefers tRNA demethylation rather than mRNA or rRNA [29]. Considering the subtle effect of ALKBH5 on mRNA and m6A dynamic regulation of rRNA or other non-coding RNA, it is unsurprising that other unknown members of the ALKB family exist.

### FTO

As FTO is able to demethylate the N6-position of A, FTO is the first enzyme associated with m6A demethylation. However, now, we prefer to believe that FTO is a demethylase for m6Am rather than m6A based on current findings. Firstly, several researches shook the connection between FTO and m6A. FTO did not show a preference for consensus site of m6A [11] whereas a strong preference for m6Am [30]. Additional indication was the low catalytic rate of FTO for m6A while its catalytic rate towards m6Am was much higher [30]. More importantly, FTO knockout brings on considerably increases in m6Am, with no detectable increase in m6A quantitative measurement. The counterpart is that the expression of FTO caused a reduction in m6Am, with no effect on m6A [30]. The detectable growth in m6A seen in FTO depletion may be attributed to a reactive rise of METTL14 [31]. Thus, FTO has been moving away from the core of m6A demethylation.

### ALKBH5/3

In quantitative measurements for m6A in mRNA, ALKBH5 knockdown caused increases, and ALKBH5 overexpression resulted in decreases, supporting the idea that ALKBH5 might make a significant difference in demethylation of m6A in mRNA [12]. What’s more, unlike FTO, ALKBH5 has no activity towards m6Am and appears preference for m6A [30]. ALKBH5 knockout mice are mostly normal except for defects in spermatogenesis. Additionally, the effect of ALKBH5 to m6A is subtle [12]. Thus, the development of mammals doesn’t require m6A demethylation, unless an unknown m6A demethylase exists.

The research about ALKBH3 proved this prediction. Notably, ALKBH3 shows a preference for m6A in tRNA rather than in mRNA or other non-coding RNA. ALKBH3-mediated tRNA demethylation promotes translation efficiency in cancer cells [29].

## m6A reader

The major mechanism by which m6A works is by recruiting m6A-binding proteins. Among them, the most comprehensive researches currently are about the YT521-B homology (YTH) domain-containing proteins family, including YTH-domain C1 (YTHDC1), YTH-domain C2 (YTHDC2), YTH-domain F1 (YTHDF1), YTH-domain F2 (YTHDF2) and YTH-domain F3 (YTHDF3). Recent years, many new m6A readers without YTH domain have been discovered. These readers, including eukaryotic initiation factor 3 (eIF3), Heterogeneous nuclear ribonucleoprotein family (HNRNPs), Proline rich coiled-coil 2A (Prrc2a) and Insulin-like growth factor 2 mRNA binding proteins (IGF2BPs), etc., have significantly enriched people’s understanding of the role of m6A modification in transcriptome.

### YTH domain proteins

The YTH domain was initially identified by the homology search of a human splicing factor YT521B [32]. This 100–150 residues domain, which was detected to attribute to RNA-binding function of YT521B, forms a tryptophan cage comprising two or three tryptophan residues around the methyl group of m6A [33]. Then, Rechavi and colleagues first linked this structure to the m6A-binding protein. In the m6A RNA pull-down experiment, they detected that YTH domain YTHDF2 and YTHDF3 could bind to m6A [2]. Several subsequent research groups verified this result using gel shift assay and crystallography, and have inspired more exploration of the role and mechanism of these YTH domain proteins [7, 34, 35].

YTHDC1 is a nuclear protein involved in hnRNA splicing. YTHDC1 regulates exon inclusion by interacting with trans- and cis- regulatory elements, such as facilitating serine and arginine-rich splicing factor 3 (SRSF3) or blocking SRSF10 [36]. The long non-coding RNA (lncRNA) X-inactive specific transcript(XIST) is a m6A-riched lncRNA, which mediates X chromosome silencing. YTHDC1 preferentially recognizes m6A residues on XIST and promotes XIST function [37]. Moreover, recent studies have shown that YTHDC1 interacts with SRSF3 and nuclear RNA export factor 1 (NXF1) to promote the nuclear export of m6A-mRNAs [38]. Another research on RNA metabolism shows that recombinant Methionine Adenosyltransferase II Alpha (MAT2A) m6A is read by YTHDC1, which induces SAM-mediated MAT2A mRNA degradation [39].

YTHDC2 is a putative RNA helicase, aside from the YTH domain. YTHDC2 selectively binds to a few m6A sites, especially in non-coding RNA. Additionally, YTHDC2 can enhance translation efficiency and promote targeted RNA degradation by selectively binding m6A [40–43].

YTHDF1 ~ 3 are cytoplasmic m6A readers. It was initially demonstrated that YTHDF1 binds to the m6A site surrounding the stop codon and facilitate the translation initiation machinery to enhance the translation efficiency of targeted RNAs [44]. In contrast, YTHDF2 accelerates the degradation of m6A-modified RNA by directly recruiting CCR4-NOT deadenylase complexes [45]. YTHDF3 can be regarded as a fine-tuning of the RNA accessibility of YTHDF1 and YTHDF2 because YTHDF3 can synergize with YTHDF1 to promote the translation of methylated RNA. This synergy then accelerates the degradation of mRNA by directly interacting with YTHDF2 [46, 47].

### eIF3

Generally, eukaryotic initiation factor 4 (eIF4) proteins, especially the cap-binding protein eIF4e, are required for translation initiation [48]. However, eIF3 was identified as a direct 5′UTR m6A-binding protein to initiate eIF4-independent translation [49]. About 35% of eIF3 Crosslinking and Immunoprecipitation (CLIP) sites mapped throughout the transcriptome overlap with m6A sites. Importantly, it is m6A, not m6Am that plays a part. Notably, METTL3 forms only m6A, not m6Am. Thus, 5′UTR m6A is probably an indicator of eIF3-dependent translation. A previous study showed m6A could directly bind and recruit eIF3 to the 5′UTR [49]. Recently, however, researchers have discovered a more complex and more ingenious mechanism for METTL3-mediated mRNA circulrization and passing eIF3 from 3′UTR to 5′UTR [44, 50]. Given m6A enrichment near 3′UTR [3], the latter is more convincing in the case of not ruling out the former.

### HNRNPs

Heterogeneous nuclear ribonucleoprotein A2B1 (HNRNPA2B1), heterogeneous nuclear ribonucleoprotein C (HNRNPC), and heterogeneous nuclear ribonucleoprotein G (HNRNPG) are three abundant nuclear RNA-binding proteins responsible for hnRNA processing [51].

The previous understanding was that HNRNPA2B1 was identified to be a regulator in microRNA (miRNA) processing by selectively binding to the m6A site of miRNA directly [52]. Interestingly, recent studies concluded that HNRNPA2B1 depletion has minimal effect on mature miRNA levels in the central nervous system [53, 54], in contrast to earlier studies. These newer findings have challenged the idea that HNRNPA2B1 has a general role in microRNA processing. Santangelo et al. showed that HNRNPA2B1 selectively binds to GGAG or GGCU motifs on miRNA, suggesting that there is sequence-specific miRNA sorting into exosomes. This result is consistent with the uneven distribution of m6A modification in miRNA [55].

HNRNPC and HNRNPG also regulate mRNA splicing and abundance by processing m6A-modified RNA transcripts. The m6A site of hnRNA indirectly alters the binding of HNRNPC/G to its U-tract motifs, thereby modulating mRNA abundance and splicing [56, 57]. This phenomenon is termed ‘m6A-switch’.

### Prrc2a

Wu and his colleagues discovered that Prrc2a is a novel m6A reader [58]. They found that Prrc2a deficiency in the nervous tissue leads to hypomyelination by affecting oligodendrocytes. Through transcriptome-wide analyses, they found that Prrc2a directly regulates *Olig2* expression in an m6A-dependent manner in vitro and in vivo, which is essential in oligodendrocyte development. One interesting point is that Prrc2a, with YTHDF2, compete for RNA binding. Another is that FTO, an m6A demethylase, can trigger hypomyelination by downregulating *Olig2* mRNA in an m6A-dependent manner [58]. These findings may build a dynamic m6A regulating hypomyelination model, which needs further studies.

### IGF2BPs

IGF2BPs, including IGF2BP1/2/3, also serve as a distinct family of m6A readers. Different from YTH domain proteins, IGF2BP1/2/3 recognize the m6A consensus sequence through the K homology domains. IGF2BPs binding m6A enhances the stability and translation efficiency of their targeted mRNAs, like *MYC* mRNA, under normal and stress conditions [59].

## Two approaches of effect of m6A on the transcriptome

The effect of m6A modification on the transcriptome has been continuously deepened with the research progress of the YTH-domain protein family and the discovery of other novel m6A readers. We summarize two regulatory approaches—direct approach and indirect approach—to systematically describe the possible regulatory mechanisms of m6A modification in the transcriptome.

### Direct regulatory approach

**Fig. 3:**
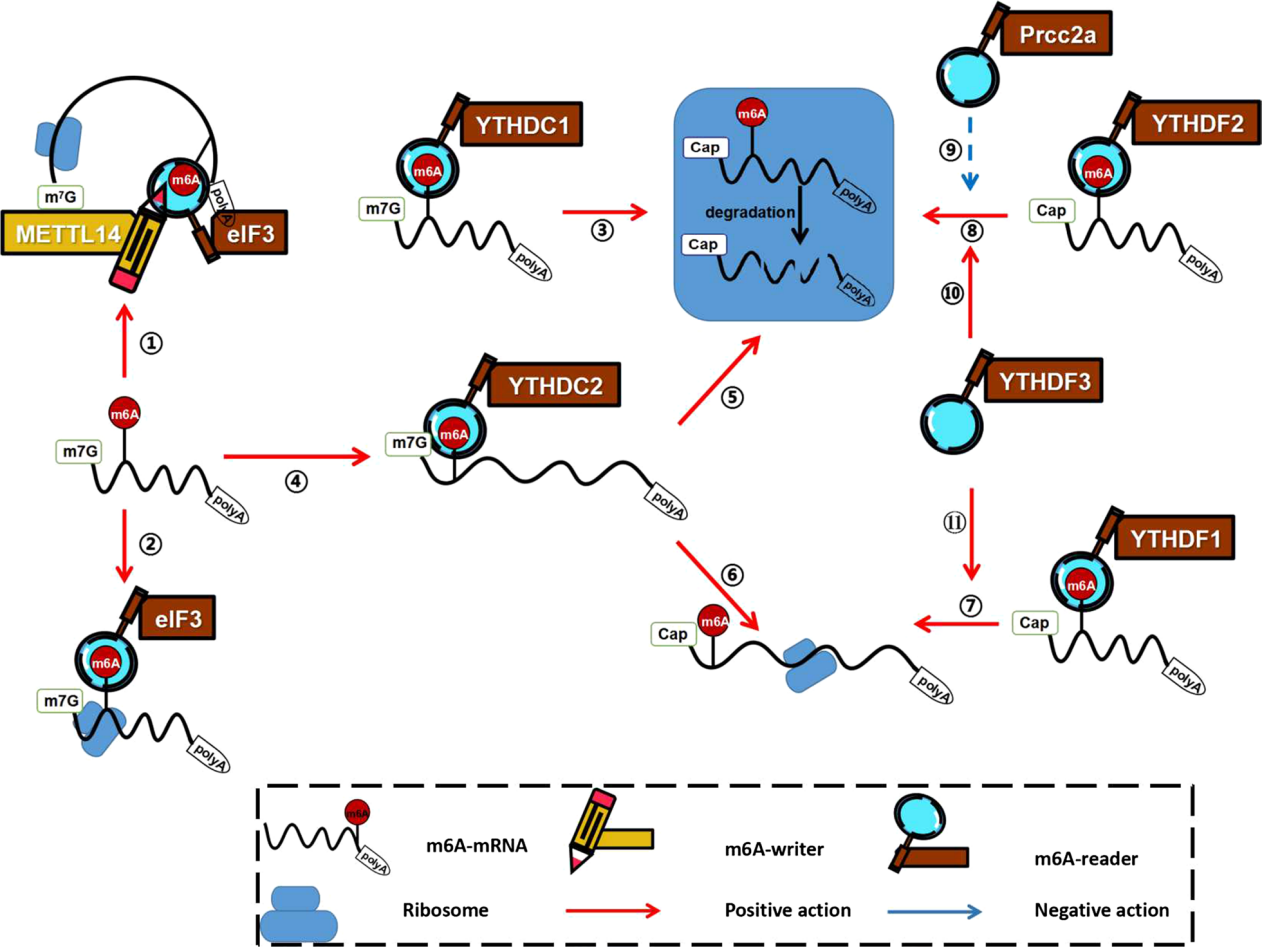
Direct pathway of m6A to regulate transcriptome **a** Two possible mechanisms of eIF3 regulating mRNA translation initiation. **①**: m6A directly promotes the recruitment of eIF3 and then induce translation initiation. **②**: m6A delivers eIF3 to the 5′UTR through METTL3–eIF3h mediated mRNA circularization and induce translation initiation. **b** Approach of YTH-domain proteins(except YTHDC1) regulating mRNA stability and metabolism. **③:** YTHDC1 reads MAT2A m6A, which induces SAM-mediated MAT2A mRNA degradation. **④ → ⑤/⑥**: YTHDC2 binds m6A and unpacks the target m6A-mRNA to enhance the translation efficiency and degradation. **⑦:** YTHDF1 binds to the m6A site surrounding the stop codon, then cooperates with the translation initiation machinery to enhance the translation efficiency of target RNAs. **⑧:** YTHDF2 accelerates the degradation of m6A-modified RNA by directly recruiting CCR4-NOT deadenylase complexes. **⑨⑩:** YTHDF3 can accelerate the degradation of mRNA by directly interacting with YTHDF2 while Prcc2a can decelerate this degradation. **⑪:** YTHDF3 can synergize with YTHDF1 to promote the translation of m6A-RNA

Some m6A readers can directly recognize m6A modification on targeted transcripts and regulate the expression of corresponding targeted proteins (Fig. 3). The direct approach includes the regulation of mRNA translation efficiency and the regulation of mRNA abundance. On the one hand, through two possible mechanisms of eIF3 initiating translation and YTHDC2 loosening the spacial structure of mRNA, m6A-mRNA translation efficiency is improved. On the other hand, loosening mRNA is easier to be degraded. YTHDC1-mediated MAT2A mRNA degradation and YTHDF2 recruiting CCR4-NOT deadenylase complexes can reduce mRNA abundance.

### Indirect regulatory approach

**Fig. 4:**
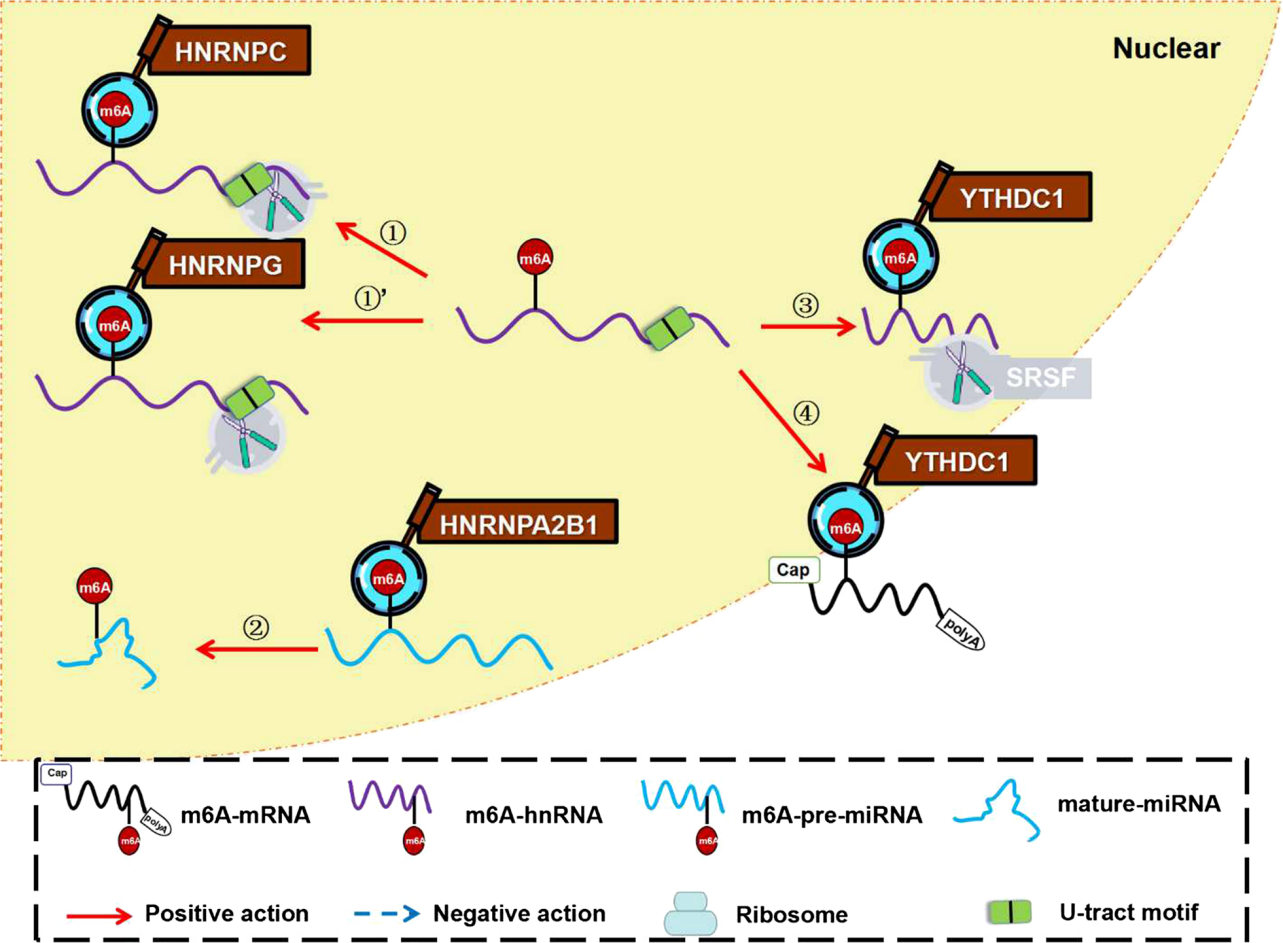
Approach of m6A readers regulating on transcriptome indirectly. **①/①’**: ‘m6A-switch’: HNRNPC and HNRNPG binding to the m6A site of hnRNA indirectly alters the connection of HNRNPC/G to its U-tract motifs, thereby modulating mRNA abundance and splicing. **②**: HNRNPA2B1 selectively binds to the m6A site of pre-microRNAs and modulates the matureness of microRNA. **③:** YTHDC1 promotes exon inclusion by recruiting or restricting different splicing factors, such as SRSFs. **④:** YTHDC1 promotes the nuclear export of m6A-methylated mRNAs

Instead of binding m6A modification on mRNA directly, the expressions of some proteins can be modulated by upstream events, such as splicing, or upstream regulators like non-coding RNA (Fig. 4). The indirect pathway mainly occurs in the nucleus and plays an important role in mRNA selective splicing, non-coding RNA maturation, and RNA nuclear export. HNRNPC and HNRNPG form ‘m6A switch’ to alter the splicing sites in U-tract motifs. HNRNPA2B1 promotes miRNA maturation. YTHDC1 regulates RNA splicing through influencing recruitment of different splicing factors and facilitates RNA nuclear export.

## Opposite roles of m6A in tumorigenesis

As we know, the effect of m6A on transcription is duplex, and through many different mechanisms, some of which are currently unknown to us. The dynamic regulation of m6A and the dynamic effects of m6A regulators determine that the overall impact of m6A on cells and tissues is specific, and m6A’s regulation of targeted protein expression also changes dynamically with changes in cell development stages.

We have compiled many studies on the effects of m6A writer, eraser, and reader on human tumor cells in recent years. We can see that the same m6A regulator can play an oncogenic role in some cancers, whereas a suppressing role in other cancers. (Table 1). Increasingly evidence showed that the levels and effects of m6A in tumor cells change continuously as the tumor progresses. For instance, changes in cellular m6A levels can alter the phenotypes of breast cancer cells [60] and the malignancy of gastric cancer [61].

**Table 1:** Opposite Roles of m6A regulators in Tumorigenesis

m6A regulator	Tumor type	Role	Key results	Ref
METTL3	Colorectal cancer	−	(1) Positive expression of METTL3 was significantly associated with longer survival time (2) METTL3 suppresses colorectal cancer proliferation and migration through p38/ERK pathways	[62]
		+	(1) METTL3 facilitates tumor progression via an m6A-IGF2BP2-dependent mechanism in colorectal carcinoma (2) METTL3 promotes CRC cell stemness by increasing the expression of SOX2	[63]
		+	METTL3 promotes metastasis of colorectal cancer via miR-1246/SPRED2/MAPK signaling pathway	[64]
	Renal clear cell carcinoma	+	(1) METTL3 might be involved in the promotion proliferation through PI3K-AKT-mTOR pathway (2) METTL3 increased cell cycle arrest in G1 phase by promoting a gain in P21 expression	[65]
		−	METTL3 inhibites cellular migration and invasion through EMT pathway	[65]
	Breast cancer	+	Hepatitis B X-interacting protein(HBXIP)-elevated METTL3 promotes the progression of breast cancer via inhibiting tumor suppressor let-7 g	[66]
		+	METTL3 promotes the breast cancer progression via targeting Bcl-2	[67]
		−	Reduced expression of METTL3 predicts poor prognosis in breast cancer	[68]
YTHDF2	Hepatocellular carcinoma	+	miR145 modulates m6A levels by targeting the 3’-UTR of YTHDF2 mRNA and is able to suppresses proliferation of hepatocellular carcinoma	[69]
		+	YTHDF2 facilitates METTL3-mediated SOCS2 m6A modification and degradation thereby reducing the tumor suppressive effect of SOCS2	[70]
		−	(1) Overexpression of YTHDF2 suppresses hepatocellular carcinoma cell proliferation and growth in vitro and in vivo (2) YTHDF2 inhibits ERK/MAPK signaling cascades by destabilizing the EGFR mRNA in hepatocellular carcinoma cells	[71]
	Pancreatic cancer	+	(1) YTHDF2 orchestrates EMT and proliferation in pancreatic cancer cells (2) YTHDF2 promotes tumor proliferation by activating AKT/GSK3β/cyclin D1	[15]
		−	YTHDF2 suppresses metastasis by destabilizing YAP mRNA	[15]

+: promoting effect; −: suppressing effect

Given the complexity of m6A’s regulation of the transcriptome, this is understandable and worth investigating its cause deeply. The contradictory results of some studies remind us that we cannot simply attribute the effects of m6A to certain tumors as oncogenic or suppressive. Therefore, we must carefully consider and pay attention to the overall impact and dynamic effects when introducing m6A modification to human cancer treatment.

## Conclusion and future prospectus

Although current research on m6A modification has not had a massive impact on the practice of cancer diagnosis and treatment, great efforts have continued.

The first hotspot is m6A-associated cancer phenotyping, staging, and prognosis. A large number of small sample studies are widely involved in various human cancers, including lung cancer [72, 73], gastric cancer [61, 74], breast cancer [60, 68], glioma [75], urogenital cancer [76] etc.

Secondly, research on anticancer treatment related to m6A is also progressing. The intervention of m6A regulators of tumor cells can become a new target for adjuvant radiotherapy and chemotherapy. Research demonstrated that the upregulation of METTL14 expression in pancreatic cancer cells could reduce the response to mTOR signal-mediated autophagy after cisplatin treatment [77]. Other studies showed that m6A writer METTL3 could promote chemo-/radio-resistance in pancreatic cancer cells [78] and glioma stem-like cells [79]. More gratifyingly, scientists have been researching drugs associated with the regulation of m6A modification. One example is the ethyl ester form of Meclofenamic acid (MA), MA2—a highly selective inhibitor of FTO [80]. MA2 suppresses glioblastoma progression and prolongs lifespan in vivo [81], which suggests that m6A methylation could be a promising target for anti-glioblastoma therapy.

Tumor mutation burden (TMB), microsatellite instability (MSI), and Epstein‐Barr virus (EBV) were recognized as markers for cancer immunotherapy. A research conducted by Zhang et al. suggested that m6A modification was positively correlated with TMB/MSI status, and might be involved in immune responses of gastric cancer [61]. EBV-associated tumorigenesis has also been confirmed to require epitranscriptome reprogramming by METTL14 [82]. Thereby exploration of novel immunotherapy strategies targeting m6A is worth in-depth.

### Future research on m6A RNA still faces many challenges

The first debate requiring to be settled is that whether those proposed functions of m6A regulators are dependent on m6A modification. Lin and colleagues identified that both wild-type and catalytically inactive METTL3 can boost the translation of some particular mRNAs such as epidermal growth factor receptor (EGFR) and the Hippo pathway effector TAZ, thus promoting the development of lung cancer [83]. Therefore, the direct causal relationship between m6A and cancer progression remains to be further established.

Another problem that needs to be solved is to clarify the selection preference of m6A. That means not only will we need to clarify the site selection mechanism of m6A modification, but also the selective binding mechanism among different m6A readers to m6A. Some new studies demonstrated that YTHDFs proteins exhibit virtually identical RNA-binding preferences at m6A sites [37]. Therefore, unknown mechanisms or new regulators that determine the selective regulation on transcriptome by m6A.

There is no doubt that much work lies ahead to acquire a complete picture of m6A epigenetics and understand how to make it benefit humanity practically.

## Data Availability

Not applicable.
